# Effectiveness and safety of bictegravir/emtricitabine/tenofovir alafenamide in people with HIV in Asia: 24-Month findings from the observational BICSTaR study

**DOI:** 10.1097/MD.0000000000047358

**Published:** 2026-01-30

**Authors:** Chia-Jui Yang, Yeon-Sook Kim, Jun Yong Choi, Chiaw Yee Choy, Po-Liang Lu, Hung-Chin Tsai, Julie Ryu, Rebecca Harrison, Jack Chang, Billy Mo, Sun Hee Lee, Chien-Ching Hung

**Affiliations:** a Department of Internal Medicine, Far Eastern Memorial Hospital, New Taipei City, Taiwan; b School of Medicine, National Yang Ming Chiao Tung University, Taipei, Taiwan; c Division of Infectious Diseases, Department of Internal Medicine, Chungnam National University School of Medicine, Daejeon, Republic of Korea; d Division of Infectious Diseases, Department of Internal Medicine, Yonsei University College of Medicine, Seoul, Republic of Korea; e Department of Infectious Diseases, National Centre for Infectious Diseases, Singapore, Singapore; f Tan Tock Seng Hospital, Singapore, Singapore; g School of Post-Baccalaureate Medicine, College of Medicine, Kaohsiung Medical University, Kaohsiung, Taiwan; h Division of Infectious Diseases, Department of Internal Medicine, Kaohsiung Medical University Hospital, Kaohsiung, Taiwan; i Section of Infectious Diseases, Department of Medicine, Kaohsiung Veterans General Hospital, Kaohsiung, Taiwan; j Global Medical Affairs, Gilead Sciences, Foster City, CA; k Real World Solutions, IQVIA, London, UK; l Medical Affairs, Gilead Sciences, Taipei, Taiwan; m Medical Affairs, Gilead Sciences, Hong Kong, Hong Kong; n Department of Internal Medicine, Pusan National University School of Medicine and Pusan National University Hospital, Busan, Republic of Korea; o Department of Internal Medicine, National Taiwan University Hospital Yunlin Branch, Yunlin, Taiwan; p Department of Tropical Medicine and Parasitology, National Taiwan University College of Medicine, Taipei, Taiwan; q Department of Internal Medicine, National Taiwan University Hospital and National Taiwan University College of Medicine, Taipei, Taiwan.

**Keywords:** antiretroviral therapy, Asia, B/F/TAF, bictegravir, real-world evidence

## Abstract

BICtegravir Single Tablet Regimen is a multiregional, observational cohort study assessing the effectiveness and safety of bictegravir/emtricitabine/tenofovir alafenamide (B/F/TAF) in treatment-naïve (TN) and treatment-experienced (TE) people with HIV. We present 24-month data for the BICtegravir Single Tablet Regimen Asia cohort. Between December 2020 and March 2024, prospective and retrospective data were collected from TN and TE people with HIV receiving B/F/TAF in routine clinical care in the Republic of Korea, Singapore, and Taiwan. Endpoints at month 24 included effectiveness (HIV-1 RNA <50 copies/mL; missing = excluded and discontinuation = failure analyses), immunological endpoints (CD4 cell count and CD4/CD8 ratio), persistence, safety, and patient-reported outcomes (prospective cohort). Overall, 334 participants (252 prospective, 82 retrospective; 66 TN, 268 TE) were included in the analysis population. Most were male (97% TN, 93% TE) with ≥1 comorbidity (56% TN, 67% TE). At month 24, HIV-1 RNA was <50 copies/mL in 91% (50/55) of TN and 97% (225/331) of TE participants (missing = excluded analysis). Median (quartile [Q]1, Q3) CD4 count increased by +260 (155, 386; *P* < .001) cells/μL in TN and +40 (−65, 150; *P* = .008) cells/μL in TE participants. Median (Q1, Q3) CD4/CD8 ratio increased by +0.38 (0.21, 0.48; *P* < .001) in TN and +0.08 (0.00, 0.19; *P* < .001) in TE participants. Patient-reported outcomes indicated an improvement in mental health and a decrease in the number of bothersome symptoms in TN participants. Persistence at month 24 was high, with 98% (60/61) of TN and 97% (251/260) of TE participants remaining on B/F/TAF. Drug-related adverse events occurred in 9% (6/66) of TN and 8% (22/268) of TE participants, leading to B/F/TAF discontinuation in <1% (3/334) of participants, all of whom were TE. B/F/TAF demonstrated high levels of effectiveness, persistence, and tolerability over 24 months in people with HIV in Asia.

## 1. Introduction

Of the estimated 39.9 million people with HIV worldwide in 2023, 6.7 million were from the Asia-Pacific region.^[1,2]^ Improvements in antiretroviral therapy (ART) and increased treatment coverage in the Asia-Pacific region have led to an increased life expectancy of people with HIV, with decreasing mortality rates.^[3–5]^ It is estimated that by 2025, people with HIV ≥50 years of age will account for nearly one-third of all people with HIV in the Asia-Pacific region.^[6]^ Older people experience higher rates of comorbidity and polypharmacy than the general population,^[7]^ and these risks are compounded in older people with HIV.^[8–12]^

Given that ART is a life-long treatment, selection of appropriate regimens is a key factor for successful long-term management of HIV.^[13,14]^ Optimal ART regimens should provide long-term virologic suppression, a high barrier to resistance, and a favorable safety profile.^[9,13,15,16]^ ART should also have a low pill burden and few drug–drug interactions to support healthy aging, and overall enable a good long-term quality of life.^[9,15,16]^ Importantly, single-tablet, once-daily ART regimens are associated with greater adherence and persistence versus multi-tablet regimens.^[17]^

Bictegravir/emtricitabine/tenofovir alafenamide (B/F/TAF) is a single-tablet ART regimen for the treatment of HIV-1. B/F/TAF has previously shown efficacy and safety in treatment-naïve (TN) and treatment-experienced (TE) people with HIV in clinical trials,^[18–24]^ including in older TE people with HIV,^[25]^ and Asian people with HIV.^[26]^ International and local guidelines across Asia recommend B/F/TAF as a first-line treatment for HIV-1,^[9,13,15]^ including in the Republic of Korea,^[27]^ Singapore,^[28]^ and Taiwan.^[29]^

A growing body of evidence supports the effectiveness and safety of B/F/TAF in real-world settings.^[30–32]^ In Taiwan, retrospective analyses of specific cohorts of people with HIV have assessed the use of B/F/TAF up to 18 months.^[31,33–35]^ Further recent studies conducted in Taiwan have assessed weight and metabolic changes in people with HIV starting on or switching to B/F/TAF,^[36,37]^ and pretreatment drug resistance trends in TN people with HIV after the introduction of bictegravir, cabotegravir, or dolutegravir.^[38]^ However, real-world data for B/F/TAF beyond 18 months are limited in the broader Asia-Pacific region.

BICtegravir Single Tablet Regimen (BICSTaR) is a prospective, multiregional, observational cohort study that aims to assess the effectiveness and safety of B/F/TAF in routine clinical care. In total, it has enrolled 2379 TN and TE people with HIV from 14 countries across 5 regional BICSTaR cohorts: Europe, Canada, Israel, Asia, and Japan.^[39]^ All participants in the main study are followed for 24 months, and the study’s primary objective is to establish the virologic effectiveness of B/F/TAF at 12 months. A pooled analysis of prospective 12-month data from 12 countries has been published,^[39]^ as have 12-month data from the BICSTaR Asia cohort,^[40]^ and individual country data from Italy,^[41]^ France,^[42]^ Canada,^[43]^ and Japan.^[44]^ The final 24-month prospective analysis from the pooled BICSTaR cohort of 14 countries has also been published recently.^[45]^

Here, we report the final 24-month effectiveness and safety analysis of prospective and retrospective data for people with HIV in the BICSTaR Asia cohort, which included participants from the Republic of Korea, Singapore, and Taiwan.

## 2. Methods

### 2.1. Study design

Detailed methods for the BICSTaR study^[39]^ and the BICSTaR Asia cohort analysis^[40]^ have been described elsewhere.

In brief, this 24-month analysis included people with HIV receiving B/F/TAF in routine clinical care at one of 14 centers across the BICSTaR Asia cohort (see Table S1, Supplemental Digital Content, https://links.lww.com/MD/R221): the Republic of Korea (n* = *7 centers), Singapore (n* = *1), and Taiwan (n* = *6). Data were collected between December 24, 2020, and March 1, 2024 (enrollment completed December 16, 2021). Prospective data were collected in TN and TE people with HIV who initiated or switched to B/F/TAF at baseline. Retrospective and prospective data were collected in TN and TE people with HIV who initiated or switched to B/F/TAF prior to baseline, herein referred to as the retrospective cohort. A subsequent amendment restricted this cohort to those on B/F/TAF for <3 months at study enrollment (site institutional review boards granted approval between February 4, 2021, and July 26, 2021). Data were collected by trained personnel at follow-up visits determined by routine clinical practice. Adverse events (AEs) were coded using the Medical Dictionary for Regulatory Activities Version 25.1.

### 2.2. Participants and treatment

People with HIV aged ≥21 years receiving B/F/TAF in routine clinical care who provided written informed consent were eligible for inclusion. Individuals with a history of participation in any interventional clinical trial (without prior approval from the study sponsor) or any observational B/F/TAF study were excluded. All participants received B/F/TAF (50/200/25 mg) in accordance with the local package information and treatment guidelines for the Republic of Korea, Singapore, and Taiwan as appropriate.^[46–48]^

### 2.3. Study endpoints and assessments

Detailed endpoints and assessments for the BICSTaR study^[39]^ and the BICSTaR Asia cohort analysis^[40]^ have been described elsewhere.

The primary endpoint was the proportion of participants with HIV-1 RNA suppression (<50 copies/mL) at month 12 (≥275 days [9 months] to ≤548 days [18 months]).^[39]^ Secondary endpoints included the proportion of participants with HIV-1 RNA <50 copies/mL at month 24 (≥549 days [18 months] to ≤913 days [30 months]), change from baseline in CD4 cell count and CD4/CD8 ratio at month 24, and the number and proportion of participants experiencing AEs, serious adverse events (SAEs), or drug-related adverse events (DRAEs) at month 24. Further exploratory endpoints were change from baseline in weight and body mass index (BMI), laboratory parameters including lipid profile, estimated glomerular filtration rate (eGFR), and blood glucose levels at month 24; reasons for initiating B/F/TAF in TN or TE participants, and treatment persistence at 24 months as measured by the proportion of participants who remained in the study having received B/F/TAF for ≥549 days. Specifically, persistence was defined as the number of participants on B/F/TAF in the 24-month visit window divided by the number of participants in the study (excluding those who discontinued the study with no evidence of stopping B/F/TAF and participants who discontinued at <549 days). In the prospective cohort, changes in several patient-reported outcomes (PROs) were measured from baseline to month 24: physical and mental health (36-Item Short-Form Health Survey [SF-36]), bothersome symptoms (HIV-Symptom Index), and treatment satisfaction (HIV Treatment Satisfaction Questionnaire-status).

### 2.4. Statistical analysis

A detailed description of the statistical analysis methods used for the BICSTaR study and the BICSTaR Asia cohort analysis has been reported elsewhere.^[39,40]^

For the BICSTaR Asia cohort, a sample size of 350 was sufficient to provide 80% power to detect a proportion of HIV-1 RNA suppression (<50 copies/mL) of ≥90%; the minimum detectable proportion was 94.1%.

The primary endpoint analysis (HIV-1 RNA suppression at month 24) was measured using a missing = excluded (M = E) analysis in participants with ≥1 HIV-1 RNA value available within the 24-month window. The TN and TE participants were analyzed separately. Participants with missing data were excluded from the M = E analysis (no imputation), as were those who discontinued the study and/or B/F/TAF before the 24-month visit window. A separate treatment discontinuation = failure (D = F) analysis was also performed. This D = F analysis included participants with ≥1 HIV-1 RNA value available within the 24-month visit window, as well as those who discontinued B/F/TAF before the lower bound of the 24-month visit window (imputed as RNA ≥50 copies/mL). Participants who discontinued B/F/TAF during the 24-month visit window were excluded from the D = F analysis (no imputation).

Demographic and outcomes data were analyzed using descriptive statistics. A 95% confidence interval was calculated for categorical and continuous variables. Changes in weight and laboratory parameters were analyzed using either the Student *t* test or sign test/Wilcoxon signed-rank test; eGFR was calculated using the Cockcroft–Gault formula. Groups with <20 participants were excluded from statistical testing. Analyses were performed using SAS software version 9.4 (SAS institute, Cary).

As described previously,^[40]^ there was potential for overestimation of persistence, virologic effectiveness, and incidence of AEs in the retrospective cohort due to immortal time bias (caused by exclusion of individuals who discontinued prior to study initiation). To reduce the impact of immortal time bias, inclusion criteria for participants entering the retrospective cohort going forwards was amended to those on B/F/TAF for <3 months at study enrollment, but existing participants were not excluded if they had initiated >3 months before enrollment. Outcomes were also stratified by cohort (retrospective vs prospective) to assess the presence of these biases.

### 2.5. Ethics approval

The protocol was approved by an institutional review board at each center (Table S1, Supplemental Digital Content, https://links.lww.com/MD/R221) and the study was conducted in accordance with Good Pharmacoepidemiology Practices, the Heads of Medicines Agencies’ Good Pharmacovigilance Practices, and the Declaration of Helsinki. All participants provided written informed consent.

## 3. Results

### 3.1. Baseline demographics and disease characteristics

In total, 342 participants were enrolled in the BICSTaR Asia cohort, with 334 (88 from the Republic of Korea, 13 from Singapore, 233 from Taiwan) eligible for inclusion in the analysis (Fig. S1, Supplemental Digital content, https://links.lww.com/MD/R222). Of the participants included, 66 were TN and 268 were TE (252 prospective and 82 retrospective). Median follow-up time was 23.5 (22.5, 24.2) months in TN participants and 23.5 (22.6, 24.5) months in TE participants; median duration of B/F/TAF treatment was 23.5 (22.5, 24.2) and 23.4 (22.4, 24.4) months, respectively.

Baseline demographics and clinical characteristics, shown in Table 1, were generally comparable across prospective and retrospective cohorts. Most participants were male (97% TN; 93% TE) and all were Asian, with TN participants younger than TE participants (median [quartile [Q]1, Q3]: 32 [28, 36] vs 42 [35, 49] years). More than half had ≥1 ongoing comorbidity (56% TN; 67% TE), with hyperlipidemia being the most common.

**Table 1:** Baseline demographics and clinical characteristics.

Characteristic	TN (n = 66)	TE (n = 268)
Sex,* n (%)		
Male	64 (97.0)	249 (92.9)
Female	2 (3.0)	19 (7.1)
Age		
Median (Q1, Q3) (yr)	31.5 (28.0, 36.0)	42.0 (35.0, 49.0)
<50 years, n (%)	58 (87.9)	203 (75.7)
≥50 years, n (%)	8 (12.1)	65 (24.3)
Median (Q1, Q3) weight (kg)	68.2 (59.9, 78.0)	69.0 (61.0, 77.3)
Median (Q1, Q3) BMI (kg/m^2^)	23.1 (21.0, 24.9)	23.8 (21.8, 25.9)
Race/ethnicity, n (%)		
Asian	66 (100)	268 (100)
Comorbidities ongoing at time of B/F/TAF initiation, n (%)		
None	29 (43.9)	88 (32.8)
1	14 (21.2)	78 (29.1)
2	12 (18.2)	55 (20.5)
≥3	11 (16.7)	47 (17.5)
Most common		
Hyperlipidemia	3 (4.5)	60 (22.4)
Hypertension	4 (6.1)	26 (9.7)
Neuropsychiatric	3 (4.5)	30 (11.2)
Osteopathic	3 (4.5)	15 (5.6)
Chronic hepatitis B	1 (1.5)	37 (13.8)
Chronic hepatitis C	1 (1.5)	18 (6.7)
HIV-1 RNA load†		
Median (Q1, Q3), log_10_ copies/mL	4.5 (4.1, 5.4)	1.3 (1.3, 1.3)
<50 copies/mL, n (%)	0	168 (91.8)
>100,000 copies/mL, n (%)	18 (31.0)	6 (3.3)
Median (Q1, Q3) CD4 count,‡ cells/µL	325 (190, 476)	590 (442, 809)
Median (Q1, Q3) CD4/CD8 ratio§	0.34 (0.20, 0.48)	0.83 (0.60, 1.09)
Late HIV diagnosis,‖ n (%)		
CD4 <350 cells/µL¶	37 (62.7)	NA
CD4 <200 cells/µL¶	23 (39.0)	NA
Concomitant non-ART medications at baseline, n (%)#		
None	45 (70.3)	132 (50.8)
1	9 (14.1)	68 (26.2)
2	3 (4.7)	32 (12.3)
≥3	7 (10.9)	28 (10.8)
Median (Q1, Q3) number of previous ART regimen**	NA	3.0 (2.0, 4.0)
Prior ART regimen (taken just prior to B/F/TAF),†† n (%)		
INSTI	NA	178 (66.4)
NNRTI	NA	67 (25.0)
PI	NA	23 (8.6)
F/TDF	NA	55 (20.5)
F/TAF	NA	172 (64.1)
History of prior virologic failure,‡‡ n (%)	NA	42 (15.7)
Time from HIV diagnosis to B/F/TAF initiation,§§ median (Q1, Q3), days	8.0 (0.0, 23.0)	NA
Median (Q1, Q3) eGFR,‖‖ mL/min/1.73 m^2^	116.1 (104.9, 139.5)	99.0 (84.1, 117.7)

ART = antiretroviral therapy, B/F/TAF = bictegravir/emtricitabine/tenofovir alafenamide, BMI = body mass index, eGFR = estimated glomerular filtration rate, F/TAF = emtricitabine/tenofovir alafenamide, F/TDF = emtricitabine/tenofovir disoproxil fumarate, INSTI = integrase strand transfer inhibitor, NA = not applicable, NNRTI = non-nucleoside reverse transcriptase inhibitor, PI = protease inhibitor, Q = quartile, TE = treatment-experienced, TN = treatment-naïve.

* Sex was defined by the participant.

† Sample size: 59 TN, 183 TE.

‡ Sample size: 57 TN, 178 TE.

§ Sample size: 56 TN, 174 TE.

‖ Missing in 7 participants, data available for TN cohort only.

¶ And/or ≥1 AIDS-defining event at baseline.

# Data were missing for 2 TN and 8 TE participants; out of a total of 132 TE participants reporting ongoing concomitant medication use, data on the number of medications was available for 128.

** Missing in 4 participants.

†† Sample size: n = 268.

‡‡ Unknown in 7 TE participants.

§§ Applicable to TN cohort only, sample size: 66.

‖‖ Sample size: 54 TN, 173 TE.

For TN participants, the most common reason for initiating B/F/TAF was early treatment according to guidelines (83%); for TE participants, the most common reason for switching to B/F/TAF was to simplify their ART regimen (66%). In total, 6% (19/334) of participants had evidence of ≥1 primary resistance mutation present at baseline (nucleoside reverse transcriptase inhibitor, 4%; non-nucleoside reverse transcriptase inhibitor, 3%; protease inhibitor, 1%), with M184V/I (3%; 10/334) and K103N/S (2%, 7/334) being the most common.

### 3.2. Virologic effectiveness

**Figure 1.**
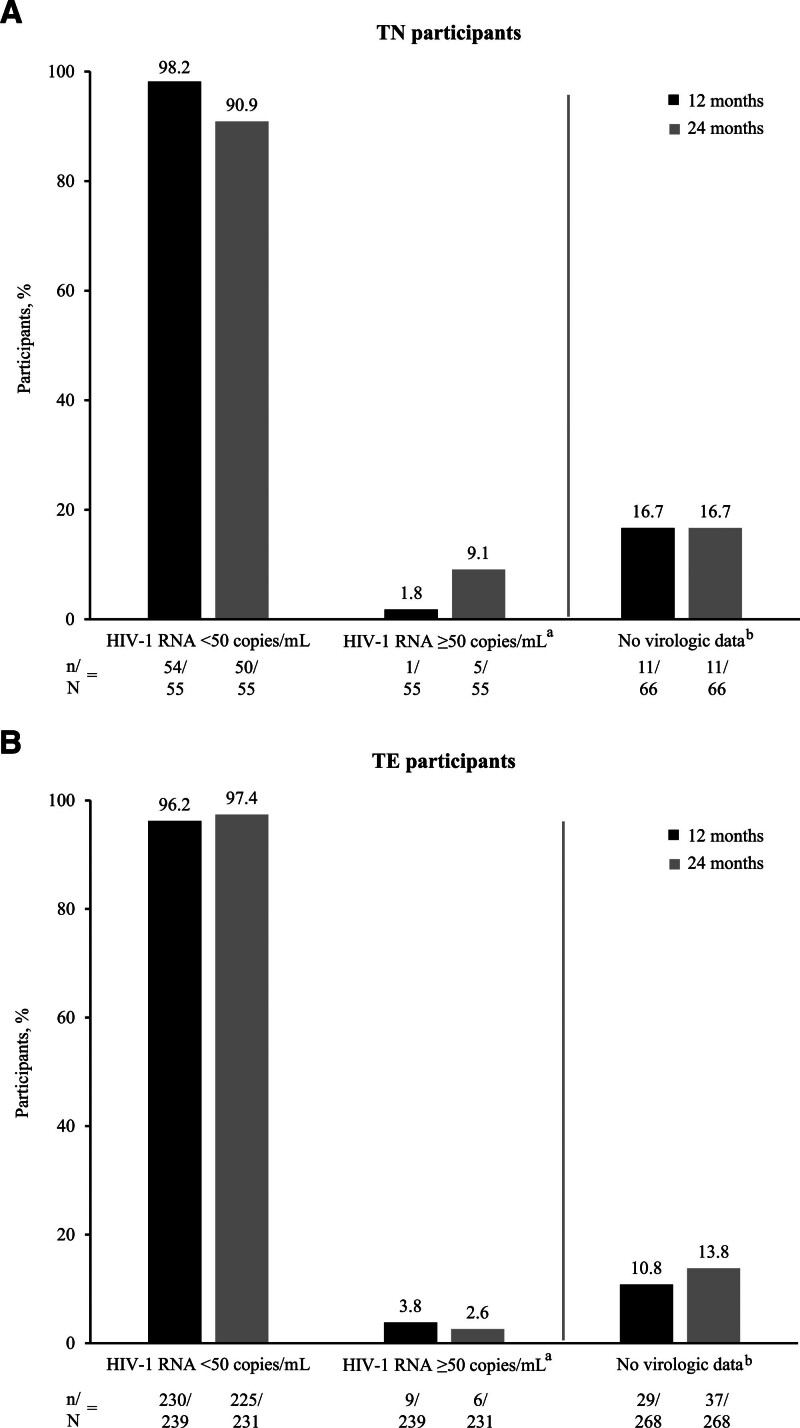
HIV-1 RNA <50 copies/mL (M = E) at 12 and 24 months in (A) TN participants and (B) TE participants. ^a^Of the 5/55 TN and 6/231 TE participants with HIV-1 RNA ≥50 copies/mL at month 24, only 1 (TN) participant had HIV-1 RNA >200 copies/mL; ^b^for the “No virologic data” category, numerators include participants with missing data and those who discontinued the study and/or B/F/TAF before the 24-month visit window (denominators represent the total analysis population of TN and TE participants). B/F/TAF = bictegravir/emtricitabine/tenofovir alafenamide; M = E = missing = excluded; TE = treatment-experienced; TN = treatment-naïve.

At month 24, the primary endpoint analysis (M = E) showed high rates of HIV-1 RNA suppression (<50 copies/mL) with B/F/TAF treatment in 91% (50/55) of TN and 97% (225/231) of TE participants (Fig. 1A and B). The D = F analysis showed HIV-1 RNA suppression in 89% (50/56) of TN and 93% (225/242) of TE participants at month 24 (Fig. S2, Supplemental Digital content, https://links.lww.com/MD/R222). There were no significant differences in HIV-1 RNA suppression between prospective and retrospective cohorts (Fig. S3, Supplemental Digital content, https://links.lww.com/MD/R222). Five participants in the TN cohort and 6 in the TE cohort had HIV-1 RNA ≥50 copies/mL at month 24 (M = E). Of these, 1 TN participant had HIV-1 RNA >200 copies/mL at month 24; most other TN and TE participants had HIV-1 RNA <100 copies/mL, except for 1 TE participant who had HIV-1 RNA 103 copies/mL. No evidence of immortal time bias (whereby the exclusion of participants with treatment failure prior to enrollment led to an overestimation of effectiveness) was found in the retrospective cohort.

### 3.3. Immunological endpoints

**Figure 2.**
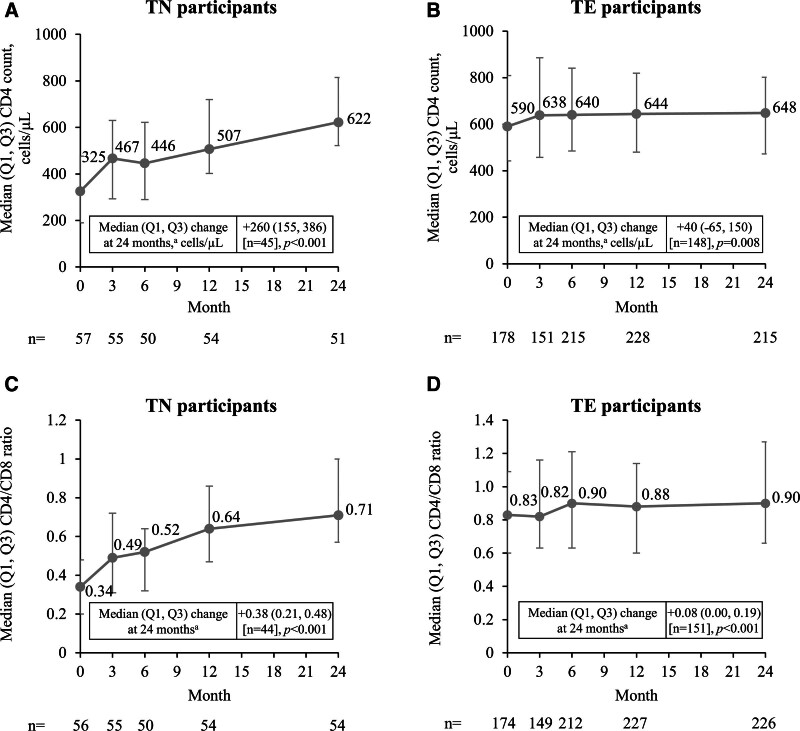
Change in CD4 cell count (A and B) and CD4/CD8 ratio (C and D) from baseline to 24 months. ^a^Median change was calculated in participants with data at baseline and 24 months; *P*-values were calculated using the sign test. Q = quartile; TE = treatment-experienced; TN = treatment-naïve.

Median (Q1, Q3) CD4 cell count increased from baseline to month 24 in both TN (+260 [155, 386] cells/μL; *P* < .001) and TE (+40 [−65, 150] cells/μL; *P* = .008) participants (Fig. 2A and B). Median (Q1, Q3) CD4/CD8 ratios increased from baseline to month 24 in TN (+0.38 [0.21, 0.48]; *P* < .001) participants and remained stable in TE (+0.08 [0.00, 0.19]; *P* < .001) participants (Fig. 2C and D). Changes in CD4 cell count and CD4/CD8 ratio were similar between prospective and retrospective cohorts.

### 3.4. Safety and tolerability

At month 24, 79% (263/334) of participants had experienced ≥1 AE (88% [58/66] of TN and 77% [205/268] of TE participants), while 10% (32/334) experienced ≥1 SAE (9% [6/66] of TN and 10% [26/268] of TE participants) (Table 2). The numbers of participants who experienced AEs or SAEs were similar between prospective and retrospective cohorts.

**Table 2:** Adverse events reported through 24 months.

Adverse events, n (%)	All (n = 334)	TN (n = 66)	TE (n = 268)
Participants with ≥1 AE	263 (78.7)	58 (87.9)	205 (76.5)
Participants with ≥1 DRAE	28 (8.4)	6 (9.1)	22 (8.2)
Gastrointestinal disorders	10 (3.0)	1 (1.5)*	9 (3.4)†
Weight gain	9 (2.7)	1 (1.5)	8 (3.0)‡
Psychiatric disorders	5 (1.5)	1 (1.5)§	4 (1.5)‖
Dizziness	4 (1.2)	1 (1.5)	3 (1.1)
Headache	1 (0.3)	0	1 (0.4)
Overweight	2 (0.6)	2 (3.0)	0
Tinnitus	1 (0.3)	1 (1.5)	0
Hiccups	1 (0.3)	0	1 (0.4)
Urticaria	1 (0.3)	1 (1.5)	0
Participants with ≥1 SAE	32 (9.6)	6 (9.1)	26 (9.7)
Any SAE related to B/F/TAF	0	0	0
Discontinuations of B/F/TAF due to DRAEs	3 (0.9)	0	3 (1.1)
Weight gain	3 (0.9)	0	3 (1.1)
Deaths	0	0	0

AE = adverse event, ART = antiretroviral therapy, B/F/TAF = bictegravir/emtricitabine/tenofovir alafenamide, DRAE = drug-related adverse event, EFV = efavirenz, SAE = serious adverse event, TDF = tenofovir disoproxil fumarate, TE = treatment-experienced, TN = treatment-naïve.

* Diarrhea.

† Constipation, diarrhea, abdominal distension, abdominal pain, flatulence, gastritis, gastroesophageal reflux disease, irritable bowel syndrome, salivary gland calculus.

‡ ART regimen just prior to B/F/TAF initiation: contained TDF (n = 7), contained EFV (n = 5).

§ Insomnia.

‖ Depressed mood, depression, poor quality sleep, sleep disorder.

DRAEs occurred in 8% (28/334) of participants, and most were mild or moderate. DRAE occurrence rates between TN (9% [6/66]) and TE (8% [22/268]) participants were similar, as were rates in prospective and retrospective cohorts. The most common DRAEs were weight-related DRAEs (n = 11; including weight gain [n = 9] and overweight [n = 2]), gastrointestinal disorders (n = 10), psychiatric disorders (n = 5), and nervous system disorders (n = 5, including 1 severe DRAE of dizziness). None of the participants reporting a nervous system disorder DRAE had an ongoing nervous system disorder at baseline.

No SAEs were related to B/F/TAF and no deaths occurred.

### 3.5. Weight change and laboratory parameters

**Figure 3.**
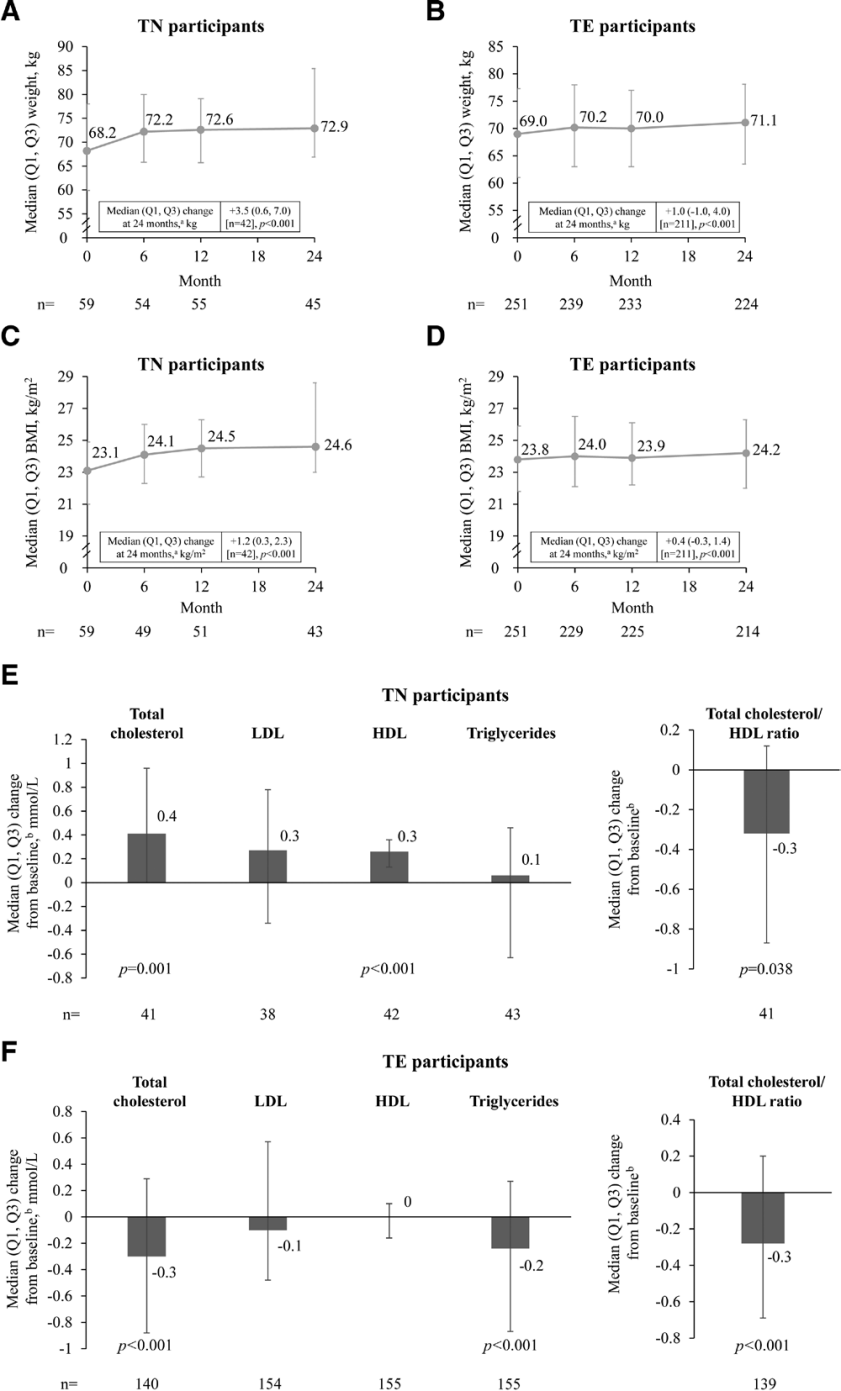
Change in weight (A and B), BMI (C and D), and lipid profile (E and F) from baseline to 24 months. ^a^Median change was calculated in participants with weight and BMI available at baseline and 24 months; *P*-values were calculated using the sign test. ^b^*P*-values determined by the sign test. BMI = body mass index; HDL = high-density lipoprotein; LDL = low-density lipoprotein; Q = quartile; TE = treatment-experienced; TN = treatment-naïve.

Through month 24, TN participants experienced a statistically significant increase in median (Q1, Q3) weight (+3.5 [0.6, 7.0] kg; *P* < .001) and BMI (+1.2 [0.3, 2.3] kg/m^2^; *P* < .001; Fig. 3A and C). TE participants also experienced small increases in median (Q1, Q3) weight (+1.0 [−1.0, 4.0] kg; *P* < .001) and BMI (+0.4 [−0.3, 1.4] kg/m^2^; *P* < .001); Fig. 3B and D). Weight increase was higher in the retrospective (+6.5 [2.3, 9.8] kg [n = 16]) than the prospective cohort (+2.5 [−0.2, 6.3] kg; *P* = .003 [n = 26]) for TN participants; however, low participant numbers prohibited statistical analysis of the difference between the retrospective and prospective groups. Weight increase was similar in retrospective and prospective TE participants (+2.0 [−0.2, 4.4] kg; *P* = .036 [n = 29] and +0.9 [−1.1, 3.9] kg; *P* = .007 [n = 182], respectively; *P* = .218 for the comparison between the retrospective and prospective groups). Weight increase was greater in TE participants with prior tenofovir disoproxil fumarate (TDF) use (+2.8 [0.7, 5.7] kg; *P* < .001) than TDF-naïve participants (+0.4 [−1.1, 3.6] kg; *P* = .012; Fig. S4, Supplemental Digital content, https://links.lww.com/MD/R222).

Of the participants who experienced weight gain coded as a DRAE (n = 9), almost all (n = 8) were in the TE cohort. Of these, 7/8 had received TDF and 5/8 had received efavirenz (EFV) as part of their ART prior to B/F/TAF initiation. In participants with available data, median (Q1, Q3) baseline weight (n = 8) was 60.9 (53.1, 72.8) kg and weight change at month 24 (n = 7) was +6.9 (−1.5, 10.6) kg; median (Q1, Q3) baseline BMI (n = 8) was 22.0 (19.5, 24.9) kg/m^2^ and BMI change at month 24 (n = 7) was +2.4 (−0.5, 3.6) kg/m^2^. For the 1 TN participant who experienced weight gain as a DRAE, baseline weight was 74.0 kg and weight change at month 24 was +9.6 kg; baseline BMI was 23.5 kg/m^2^ and BMI change at month 24 was +3.0 kg/m^2^. For the 2 TN participants who experienced a DRAE coded as overweight, baseline weight was 74.7 kg and 72.6 kg, respectively, weight change at month 24 was +10.0 kg and +10.9 kg; baseline BMI was 26.2 kg/m^2^ and 24.1 kg/m^2^, respectively, BMI change at month 24 was +3.5 kg/m^2^ and +3.6 kg/m^2^.

Small but statistically significant and clinically meaningful changes from baseline were observed in lipid profiles (Fig. 3E and F). High-density lipoprotein (HDL) cholesterol levels increased (median [Q1, Q3] change: +0.26 [0.13, 0.36] mmol/L; *P* < .001) in TN participants, while total cholesterol to HDL cholesterol ratios decreased in both TN (−0.32 [−0.87, 0.12]; *P* = .038) and TE (−0.28 [−0.69, 0.20]; *P* < .001) participants (Fig. 3E and F).

There were small but statistically significant reductions in median (Q1, Q3) eGFR levels in both TN (−9.4 [−15.8, −2.2] mL/min/1.73 m^2^; *P* < .001) and TE (−4.1 [−11.6, 2.0] mL/min/1.73 m^2^; *P* < .001) participants at month 24 (Fig. S5, Supplemental Digital content, https://links.lww.com/MD/R222); however, these reductions were not deemed clinically meaningful. For TE participants, there was a small but statistically significant reduction from baseline in blood glucose (median [Q1, Q3] −0.11 [−0.5, 0.2] mmol/L; *P* = .026) at month 24 (Fig. S6, Supplemental Digital content, https://links.lww.com/MD/R222). This decrease was present at month 6 and remained stable from month 6 to month 24.

### 3.6. Persistence and study drug discontinuations

Persistence remained high at month 24, with 98% (60/61) of TN and 97% (251/260) of TE participants remaining on B/F/TAF. Persistence was similar in prospective and retrospective cohorts. One TN participant discontinued due to participant decision (Table 3). Of the TE participants, 6 discontinued due to participant decision, 3 due to DRAEs (weight gain), and 2 due to lack of effectiveness.

**Table 3:** Reasons for B/F/TAF discontinuation through 24 months.

	TN (n = 66)	TE (n = 268)
B/F/TAF discontinuation within the 24 months following treatment initiation, n (%)	1 (1.5)	11 (4.1)
Reason for discontinuation, n (%)		
Adverse event	0	3 (1.1)*
Lack of effectiveness	0	2 (0.7)†^,^‡
Participant decision	1 (1.5)	6 (2.2)

All B/F/TAF discontinuations within the upper bound of the 24-month time window (913 days) have been considered.

ABC/DTG/3TC = abacavir/dolutegravir/lamivudine, ART = antiretroviral therapy, B/F/TAF = bictegravir/emtricitabine/tenofovir alafenamide, FTC/RPV/TAF = emtricitabine/rilpivirine/tenofovir alafenamide, TE = treatment-experienced, TN = treatment-naïve.

* Adverse events were weight gain (n = 3).

† Last on-treatment viral loads for the 2 participants were HIV-1 RNA 8490 copies/mL (month 3) and HIV-1 RNA 70 copies/mL (month 6), respectively.

‡ ART regimens switched to by participants discontinuing due to lack of effectiveness: ABC/DTG/3TC (n = 1), FTC/RPV/TAF (n = 1).

For the 3 participants who discontinued B/F/TAF because of weight gain, ART regimens taken before B/F/TAF initiation were F/TDF + nevirapine (n = 1), lamivudine/EFV/TDF (n = 1), and zidovudine/lamivudine + lopinavir/ritonavir (n = 1). For these participants (where data are available), median (Q1, Q3) baseline weight (n = 3) was 70.6 (61.6, 76.0) kg and weight change at month 24 (n = 2) was +2.9 (−1.5, 7.2) kg; median (Q1, Q3) baseline BMI (n = 3) was 24.7 (21.7, 25.1) kg/m^2^, and BMI change at month 24 (n = 2) was +1.0 (−0.5, 2.5) kg/m^2^. There were no B/F/TAF discontinuations due to renal, bone, or hepatic DRAEs.

For the 2 participants who discontinued due to lack of effectiveness, viral loads were HIV-1 RNA 2364 copies/mL at −81 days (pre-baseline) and 8490 copies/mL at 89 days, and HIV-1 RNA 70 copies/mL at −7 days (baseline) and 55 copies/mL at 203 days, respectively.

### 3.7. Patient-reported outcomes (prospective cohort only)

There was a statistically significant improvement in SF-36 Mental Component Summary (MCS) scores in TN participants (median [Q1, Q3] +2.8 [−3.1, 14.6]; *P* = .020) at month 24 (Table 4). SF-36 Physical Component Summary (PCS) scores remained stable through month 24 in TN participants (Table 4). Both PCS and MCS scores remained stable in TE participants. HIV-Symptom Index bothersome symptom count showed a statistically significant decrease from baseline at month 24 in TN participants (median [Q1, Q3] −2.0 [−5.0, −1.0]; *P* < .001) and remained stable in TE participants. HIV Treatment Satisfaction Questionnaire-status scores were high among TE participants at baseline (median [Q1, Q3] 57 [52, 60]) and remained stable at month 24 (59 [55, 60]).

**Table 4:** PROs at baseline and change from baseline at 24 months (prospective cohort only).*

	TN (n = 66)	TE (n = 268)
**SF-36 MCS score** †		
Baseline		
n	32	200
Median (Q1, Q3) score†	41.8 (31.8, 52.7)	47.8 (42.4, 52.6)
24 months		
n	32	200
Median (Q1, Q3) change from baseline	+2.8 (−3.1, 14.6)	+1.1 (−3.4, 5.5)
*P*-value for change from baseline	*P* = .020	*P* = .055
**SF-36 PCS score** †		
Baseline		
n	32	200
Median (Q1, Q3) score	56.5 (49.3, 58.7)	57.4 (53.6, 59.9)
24 months		
n	32	200
Median (Q1, Q3) change from baseline	+0.3 (−6.0, 5.3)	+0.2 (−2.7, 2.9)
*P*-value for change from baseline	*P* = .860	*P* = .358
**HIV-SI overall bothersome symptom count** ‡		
Baseline		
n	32	200
Median (Q1, Q3) overall score	5.0 (2.5, 9.5)	2.0 (0.0, 5.0)
24 months		
n	32	200
Median (Q1, Q3) change from baseline	−2.0 (−5.0, −1.0)	0.0 (−2.0, 1.0)
*P*-value for change from baseline	*P* < .001	*P* = .936

HIV-SI = HIV-Symptom Index, MCS = Mental Component Summary, PCS = Physical Component Summary, PRO = patient-reported outcome, Q = quartile, SF-36 = 36-item Short-Form Health Survey, TE = treatment-experienced, TN = treatment-naïve.

* Participants with score available at baseline and 24 months.

† Median scores >50 indicate better than average function; *P*-values were calculated using the Student *t* test for MCS and the sign test for PCS scores.

‡ Bothersome symptom count ranges from 0 to 20, with a higher count indicating more bothersome symptoms; *P*-values were calculated using the sign test.

## 4. Discussion

Until now, there have been limited real-world effectiveness and safety data for B/F/TAF use beyond 18 months in people with HIV in the Asia-Pacific region. To the best of our knowledge, this analysis provides the first 24-month real-world effectiveness and safety data for people with HIV in Asia taking B/F/TAF in routine clinical practice, supporting previously published 24-month and 12-month multiregional data from the pooled BICSTaR cohort,^[39,45]^ 12-month data from the Asia cohort,^[40]^ and analyses from Italy,^[41]^ France,^[42]^ Canada,^[43]^ and Japan.^[44]^

As previously observed,^[40]^ compared with the pooled (multiregional) population,^[39]^ BICSTaR participants from the Asia cohort had a lower median age, a lower median baseline CD4 count, a lower median body weight, and were more likely to be male. B/F/TAF maintained high levels of HIV-1 RNA suppression (≥91%) through 24 months in this Asian cohort, consistent with 24-month findings from the pooled cohort (≥94%).^[45]^ Improvements in CD4 cell count and CD4/CD8 ratio continued to demonstrate restoration of immunological function in TN participants and maintenance in TE participants. Persistence remained high (≥97%), suggesting that B/F/TAF remains well tolerated at 24 months, an important consideration given that ART is a long-term, life-long treatment for people with HIV in Asia and elsewhere. No new safety signals were observed at 24 months.

Most of the weight gain observed at month 24 was previously observed at month 12.^[40]^ The weight gain in TN participants was consistent with the increase observed following the initiation of other ART regimens in Asian people with HIV.^[49]^ Others have reported that the choice of ART has little impact on weight gain,^[50]^ which may instead be associated with a “return to health” effect.^[51]^ The weight gain observed in TE individuals in this analysis was similar to the small but statistically significant increase observed in a recent retrospective observational cohort study of Asian people with HIV who switched to B/F/TAF,^[37]^ and is consistent with the average adult annual weight gain of 0.5 to 1 kg per year.^[52]^ In addition, physical inactivity and a change in diet during the COVID-19 pandemic may have contributed to regional average weight gain.^[53]^ Of note, in this study, the majority of TE participants who experienced a DRAE of weight gain had switched to B/F/TAF from an ART regimen containing TDF or EFV, both of which are associated with weight-suppressive effects.^[54]^ The weight gain observed with the use of B/F/TAF in this analysis was accompanied by an increase in HDL cholesterol, which is generally associated with less risk of cardiovascular disease than low-density lipoprotein cholesterol.^[55,56]^ Some small but statistically significant changes were also observed in total cholesterol and triglycerides, as well as changes in total cholesterol to HDL cholesterol ratios.

Optimized health-related quality of life is a key pillar of long-term healthy aging in people with HIV.^[16]^ In this analysis, statistically significant increases were observed in MCS scores at 24 months in TN participants, indicating an improvement in mental health. A statistically significant reduction in the number of bothersome symptoms was also observed in TN participants. For TE participants (who had switched to B/F/TAF from another ART regimen), PCS scores, MCS scores, and bothersome symptom counts remained stable over 24 months. HIV Treatment Satisfaction Questionnaire-change data were not collected at 24 months for most participants in this study; however, it is notable that available HIV Treatment Satisfaction Questionnaire-change scores showed a statistically significant increase at 12 months in TE participants, indicating an improvement in treatment satisfaction compared with their previous ART regimen.^[40]^ Taken together, these PRO data are reassuring for the overall and long-term health of people with HIV as they age, particularly given the increasing proportion of older people with HIV in the Asia-Pacific region.^[6]^

Study limitations for the BICSTaR Asia cohort analyses have been described elsewhere.^[40]^ As an observational cohort study, these include a lack of randomization, as well as selection and information bias, mitigated by clearly defined eligibility criteria and the use of standardized electronic case report forms. Retrospective data should be interpreted with caution due to the risk of immortal time bias, however, no evidence of this was found here. Although this three-country analysis might not be representative of the entire Asia-Pacific region, a shared BICSTaR protocol across global cohorts allows comparison with diverse populations.

## 5. Conclusions

In this final 24-month analysis from the BICSTaR Asia cohort, B/F/TAF demonstrated high levels of effectiveness and persistence among people with HIV receiving routine clinical care in the Republic of Korea, Singapore, and Taiwan. B/F/TAF was well tolerated, with a favorable safety and resistance profile through 24 months of treatment, and no new safety signals were detected. PROs improved or remained stable, indicating that B/F/TAF maintained or improved quality of life. These findings support the long-term use of B/F/TAF for people with HIV in Asia.

## Acknowledgments

We thank all participants and investigators involved in the BICSTaR Asia cohort. Medical writing support, including development of a draft outline and subsequent drafts in consultation with the authors, collating author comments, copyediting, fact-checking, and referencing, was provided by Jack Seymour, BSc, and Josh Lilly, PhD, CMPP, at Aspire Scientific Limited (Manchester, UK). Funding for medical writing support for this article was provided by Gilead Sciences (Taipei City, Taiwan). Additional correspondence for the Republic of Korea: Sun Hee Lee, Department of Internal Medicine, Pusan National University School of Medicine and Pusan National University Hospital, Busan 49241, Republic of Korea; email: zzanmery@gmail.com; tel: +82 512407673.

## Author contributions

**Conceptualization:** Julie Ryu.

**Formal analysis:** Rebecca Harrison.

**Investigation:** Chia-Jui Yang, Yeon-Sook Kim, Jun Yong Choi, Chiaw Yee Choy, Po-Liang Lu, Hung-Chin Tsai, Sun Hee Lee, Chien-Ching Hung.

**Writing – review & editing:** Chia-Jui Yang, Yeon-Sook Kim, Jun Yong Choi, Chiaw Yee Choy, Po-Liang Lu, Hung-Chin Tsai, Julie Ryu, Rebecca Harrison, Jack Chang, Billy Mo, Sun Hee Lee, Chien-Ching Hung.

## Supplementary Material




