# Supplementary material for: Effectiveness and safety of bictegravir/emtricitabine/tenofovir alafenamide in people with HIV in Asia: 24-Month findings from the observational BICSTaR study
Source: Medicine (Baltimore). 2026 Jan 30;105(5):e47358. doi: 10.1097/MD.0000000000047358 (PMC12863871; doi:10.1097/MD.0000000000047358)
Supplement: Supplementary file 1 [file medi-105-e47358-s001.pdf]

Table S1. List of institutional review boards (IRBs).

Site ID	Site Name	IRB Name
14269	Korea University Ansan Hospital, Republic of Korea	Korea University Ansan Hospital Institutional Review Board
14270	Chungnam National University Hospital, Republic of Korea	Chungnam National University Hospital Institutional Review Board
14272	Kyungpook National University Hospital, Republic of Korea	Kyungpook National University Hospital Institutional Review Board
14277	Ajou University Hospital, Republic of Korea	Ajou University Hospital Institutional Review Board
14285	Severance Hospital, Yonsei University Health System, Republic of Korea	Yonsei University Health System, Severance Hospital, Institutional Review Board
15388	Pusan National University Hospital, Republic of Korea	Pusan National University Hospital Institutional Review Board
15447	Soon Chun Hyang University Hospital Seoul, Republic of Korea	Soon Chun Hyang University Hospital Seoul Institutional Review Board
18598	National Centre for Infectious Diseases, Singapore	National Health Group Domain Specific Review Board Domain E
14903	National Taiwan University Hospital, Taiwan	Research Ethics Committee D National Taiwan University Hospital
15247	Kaohsiung Medical University Chung-Ho Memorial Hospital, Taiwan	Kaohsiung Medical University Chung-Ho Memorial Hospital Institutional Review Board
15249	Far Eastern Memorial Hospital, Taiwan	Research Ethics Review Committee Far Eastern Memorial Hospital
15250	Taoyuan General Hospital, Taiwan	Medical Ethics and Institutional Review Board of Taoyuan General Hospital, Ministry of Health and Welfare
17935	Kaohsiung Veterans General Hospital, Taiwan	Kaohsiung Veterans General Hospital Institutional Review Board
19516	Changhua Christian Hospital, Taiwan	Changhua Christian Hospital Institutional Review Board
