# Supplementary material for: Effectiveness and safety of bictegravir/emtricitabine/tenofovir alafenamide in people with HIV in Asia: 24-Month findings from the observational BICSTaR study
Source: Medicine (Baltimore). 2026 Jan 30;105(5):e47358. doi: 10.1097/MD.0000000000047358 (PMC12863871; doi:10.1097/MD.0000000000047358)

**Figure S1.** Participant flow diagram.

^a^More than one reason may apply for one participant.

^b^For example, inclusion of ineligible subject.

In TN participants, the median (Q1, Q3) duration of B/F/TAF treatment was 23.5 (22.5, 24.3) months and 23.3 (22.5, 24.2) months in the retrospective and prospective groups, respectively; for TE participants, these values were 23.0 (22.1, 24.0) months and 23.5 (22.6, 24.5) months, respectively.

B/F/TAF = bictegravir/emtricitabine/tenofovir alafenamide; Q = quartile; TE = treatment-experienced; TN = treatment-naïve.


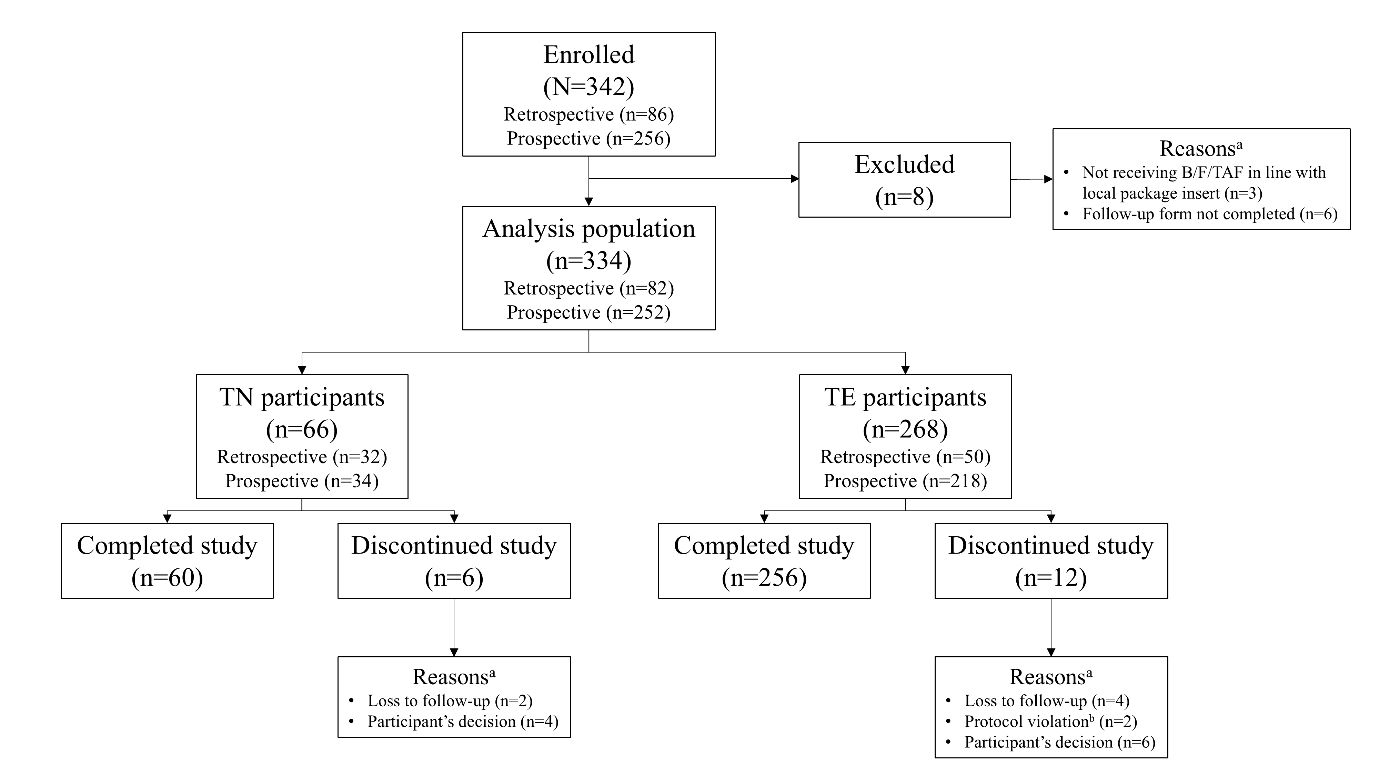


**Figure S2.** HIV-1 RNA <50 and >50 copies/mL (D=F) at 12 and 24 months in (**A**) TN and (**B**) TE participants.

D=F = treatment discontinuation = failure; TE = treatment-experienced; TN = treatment-naïve.


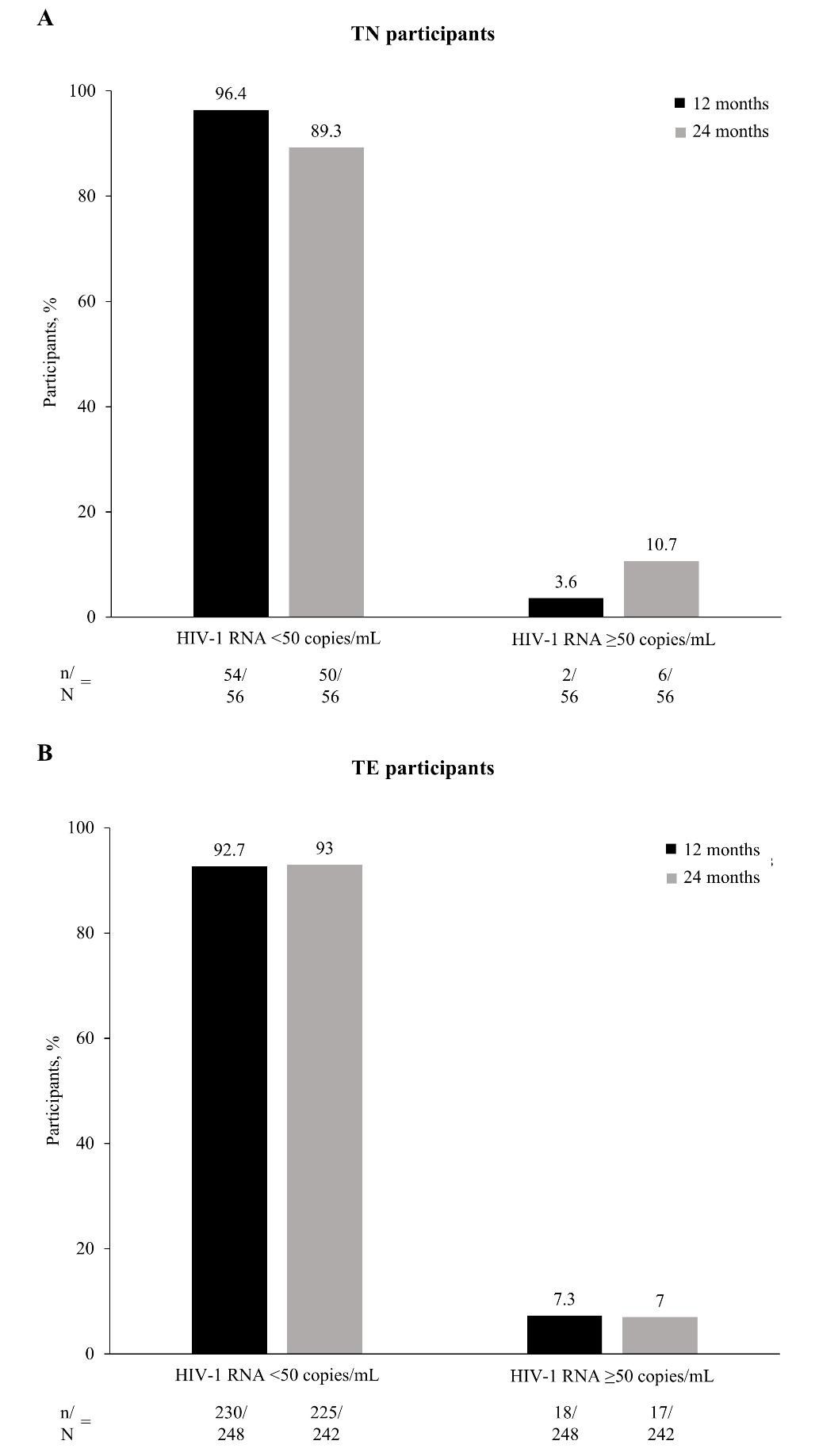


**Figure S3.** HIV-1 RNA <50 and >50 copies/mL (M=E) at 24 months (retrospective and prospective cohorts) for (**A**) TN and (**B**) TE participants.

M=E = missing = excluded; TE = treatment-experienced; TN = treatment-naïve.
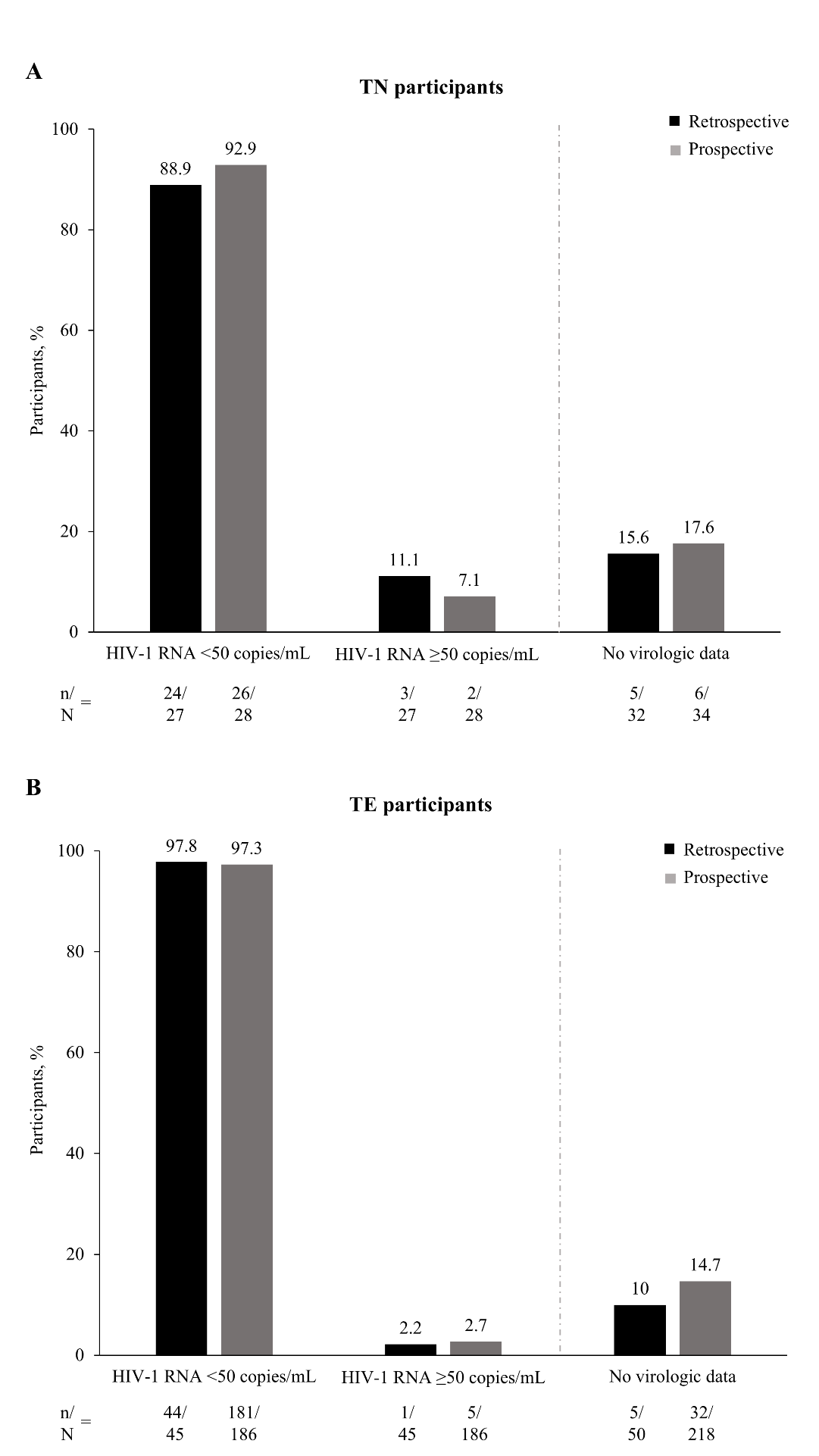


**Figure S4.** Change in (**A**) weight and (**B**) BMI from baseline to 24 months by prior TDF-containing regimen.

^a^Participants with weight data at baseline and 24 months, and data on prior TDF use.

^b^Participants with BMI data at baseline and 24 months, and data on prior TDF use. BMI = body mass index; Q = quartile; TDF = tenofovir disoproxil fumarate; TE = treatment-experienced; TN = treatment-naïve.
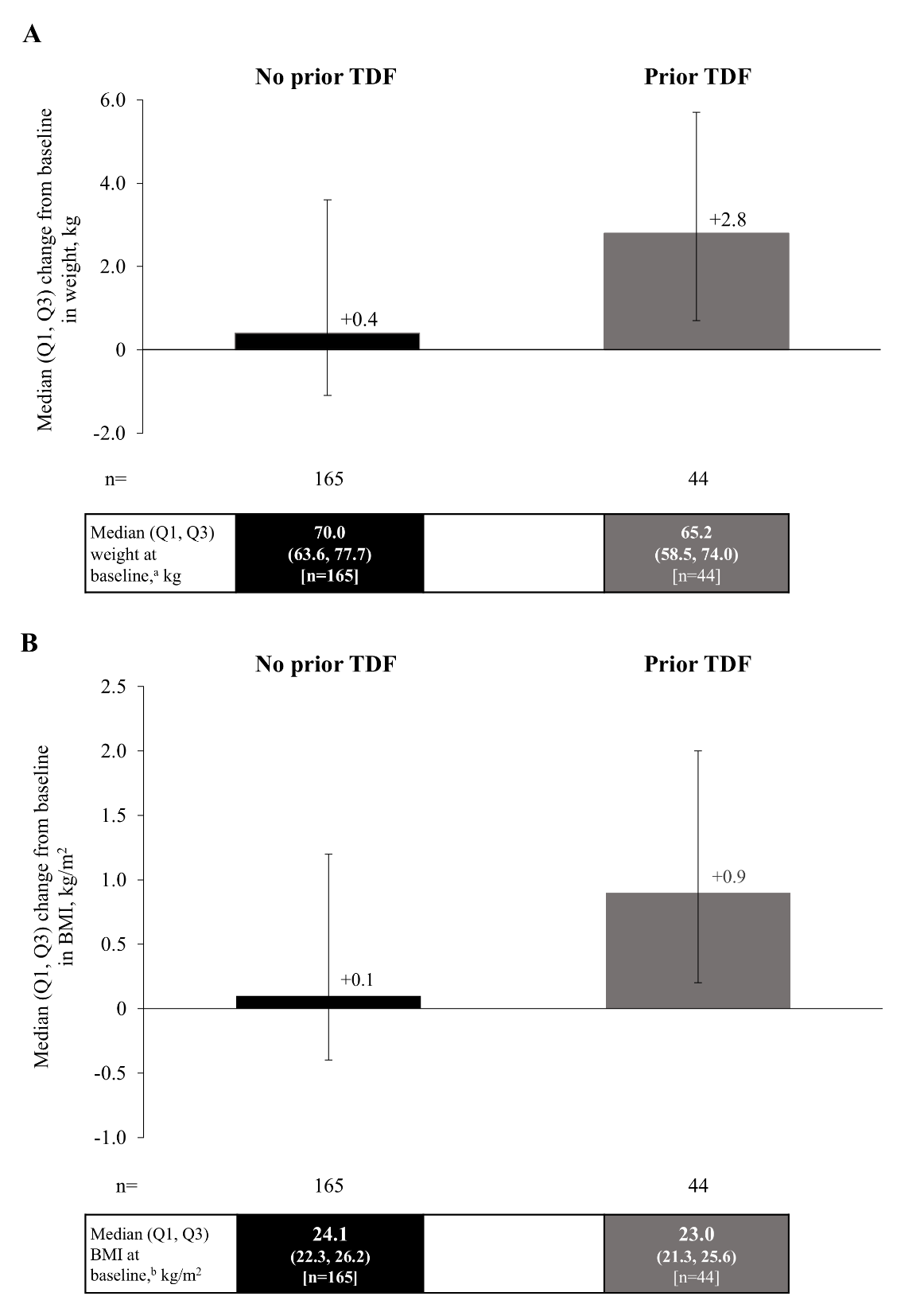


**Figure S5.** Change in eGFR levels from baseline to 24 months in (**A**) TN and (**B**) TE participants.

^a^Median change was calculated in participants with data at baseline and 24 months. *P-*values determined using the signed-rank test.

eGFR = estimated glomerular filtration rate; Q = quartile; TE = treatment-experienced; TN = treatment-naïve.


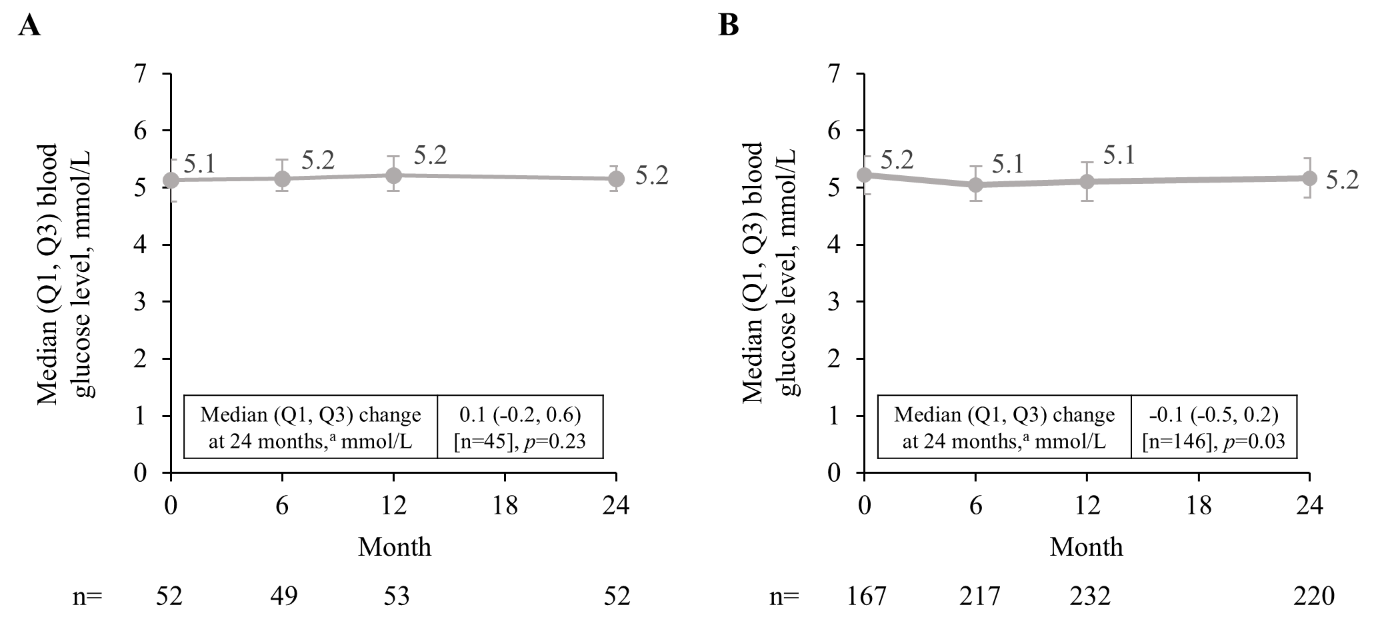


**Figure S6.** Change in blood glucose level from baseline to 24 months in (**A**) TN and (**B**) TE participants.

^a^Median change was calculated in participants with data at baseline and 24 months. *P-*values determined using the sign test.

Q = quartile; TE = treatment-experienced; TN = treatment-naïve.


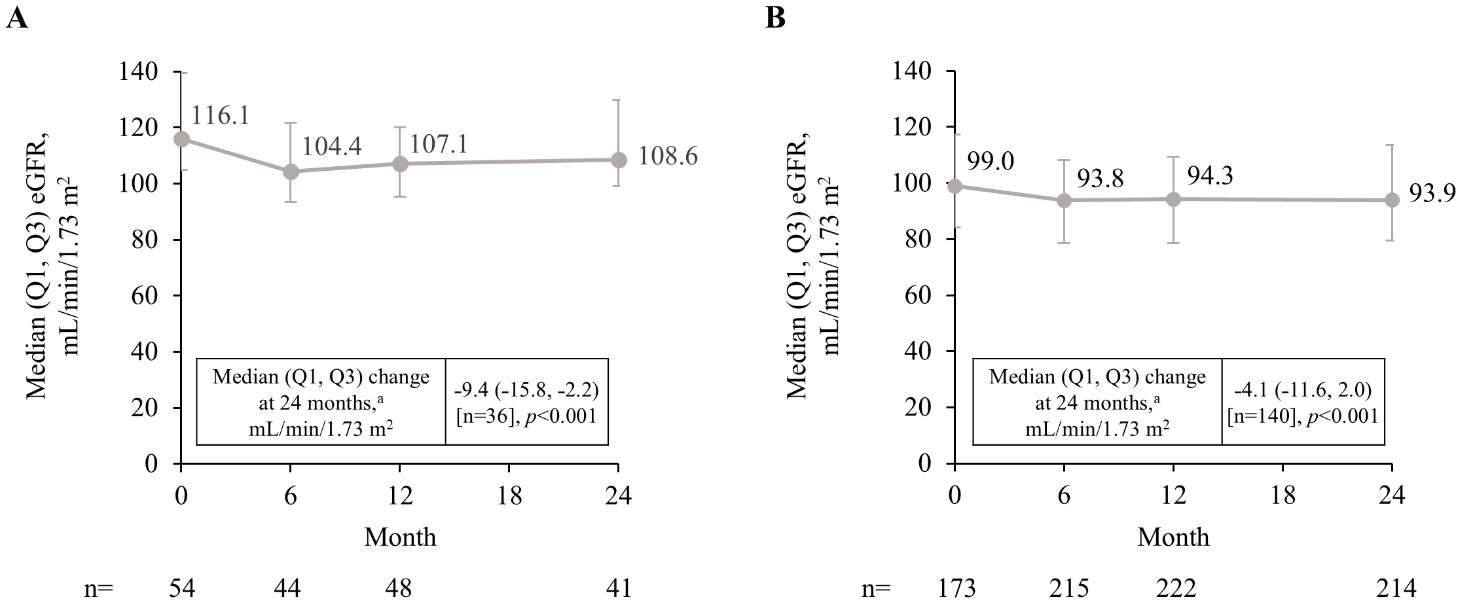

Supplement: Supplementary file 2 [file medi-105-e47358-s002.docx]
